# Comparative Response of *Ruditapes philippinarum* and *Mercenaria mercenaria* to Acute Heat and Hyposaline Stress

**DOI:** 10.3390/ani16081243

**Published:** 2026-04-17

**Authors:** Maolong Yi, Yujia Liu, Tao Wei, Yaoran Fan, Baojun Tang, Hanfeng Zheng

**Affiliations:** 1 Fisheries College, Zhejiang Ocean University, Zhoushan 316022, China; 18789589687@163.com; 2 East China Sea Fisheries Research Institute, Chinese Academy of Fishery Sciences, Shanghai 200090, China; liuyj2000@126.com (Y.L.); wt18020479686@163.com (T.W.); fanyrhh@163.com (Y.F.); bjtang@yeah.net (B.T.)

**Keywords:** *Ruditapes philippinarum*, *Mercenaria mercenaria*, physiological changes, heat shock proteins, antioxidant defense, fatty acid metabolism

## Abstract

This study explored the physiological responses and gene expression profiles of the Manila clam (*Ruditapes philippinarum*) and the hard clam (*Mercenaria mercenaria*) under heat and hyposaline stress. Experimental conditions involved increasing the temperature from 25 °C to 35 °C and decreasing salinity from 25 ppt to 15 ppt over a 6 h acclimation period, followed by 72 h exposure. Key physiological and immune indicators, including filtration rate, oxygen consumption rate, ammonia excretion rate, and the expression of related genes, were measured. Under heat stress, *R. philippinarum* exhibited higher filtration, oxygen consumption, and ammonia excretion rates than *M. mercenaria* at most sampling time points. The expression of fatty acid desaturase (Δ*6FAD*) and heat shock protein (*HSP70*) genes increased and then decreased for both species, whereas superoxide dismutase (*Cu*/*Zn SOD*) gene expression gradually decreased over time. Furthermore, the expression levels of all three genes were generally significantly higher in *M. mercenaria* compared to *R. philippinarum*. Under hyposaline stress, *R. philippinarum* exhibited significantly higher filtration, oxygen consumption, and ammonia excretion rates than *M. mercenaria* between 24 h and 72 h. Expression levels of the Na^+^-K^+^-ATPase (*NKAα*), *HSP70*, and *Cu*/*Zn SOD* genes remained higher in *M. mercenaria* compared to *R. philippinarum*. Overall, the present study indicates that *M. mercenaria* maintains relative stability and *R. philippinarum* exhibits greater physiological fluctuation under both heat and hyposaline stress. This study highlights bivalve species-specific responses to environmental stressors and provides valuable insights for aquaculture planning and ecological management in different environmental regions, particularly in the context of global climate change.

## Simple Summary

In recent years, variations in temperature and salinity have significantly impacted the physiological biochemistry and gene expression levels of bivalve mollusks. With the introduction of the hard clam into China, and its expanded aquaculture came the risk of its dispersal into natural marine waters. If this happens, it could threaten the existence of native bivalves through competition, highlighting the need for comparative research. This study combines physiological and biochemical indicators with gene expression analysis to investigate the physiological responses and adaptive mechanisms of the Manila clam (native) and the hard clam in response to environmental changes. It further reveals the molecular-level gene expression patterns underlying their adaptation to environmental shifts. Results provide a theoretical foundation for future selection and maintenance of marine economic bivalves, as well as insights into the protection of native bivalve species.

## 1. Introduction

The Manila clam, *Ruditapes philippinarum,* is extensively distributed along the eastern coastline of China and is a major traditional marine aquaculture bivalve species in the country. This species possesses significant economic value, with annual production exceeding three million metric tons [1]. The hard clam, *Mercenaria mercenaria*, is native to the eastern coasts of the United States and Canada and was introduced to China from the United States in 1997 by Zhang Fusui at the Institute of Oceanology, Chinese Academy of Sciences. Given its strong adaptability to variations in temperature and salinity in new habitats, *M. mercenaria* has been successfully cultured along the Chinese coast, from the Liaoning to Fujian Province [2]. As the scale of *M. mercenaria* aquaculture continues to expand in China, the risk of its dispersal into natural marine environments has increased. As both species are infaunal, filter-feeding bivalves, they occupy similar ecological niches, particularly in tidal flats, where they exhibit a preference for sandy substrates [3]. *Ruditapes philippinarum*, with an optimal growth temperature of 15 to 28 °C and an optimal salinity range of 20–30 ppt, mainly inhabits mud-sandy beaches in the intertidal zone with a temperature range of 5–35 °C and a salinity range of 16–36 ppt [4,5,6]. Although individuals can maintain normal metabolic activities after long-term exposure to 15 ppt [7], histological analyses have revealed that salinity levels below 15 ppt induce physiological and morphological abnormalities leading to increased mortality [6]. *Mercenaria mercenaria* occurs in salinities above 12 ppt, and adult clams can survive temperatures ranging from −6 °C to 45.2 °C. Optimal growth occurs at temperatures between 21 and 31 °C [3], but growth ceases when water temperatures fall below 9 °C or exceed 31 °C [8]. Its salinity tolerance ranges from 12 ppt to 48 ppt [3]. The ecological similarities and adaptability of *R. philippinarum* and *M. mercenaria* could lead to interspecific competition in both aquaculture systems and natural habitats. Therefore, comparative research on the physiological responses of these two species is of considerable significance.

Temperature is an important stress factor in marine environments, significantly affecting shellfish behavior, growth rate, physiology, and biochemical characteristics, and thus risking mass mortality events in both farmed and wild bivalve populations during summer [9]. In recent decades, cultured Manila clams have experienced severe summer mortality events [10]. Prolonged exposure to elevated temperatures can also increase mortality in *R. philippinarum* through disrupted physiological homeostasis, suppressed metabolic activity, and cellular damage [11,12]. Exposure to elevated temperature (27 °C) has been shown to increase mortality in *M. mercenaria* [13]. Notably, heat stress can also enhance susceptibility to parasitic infections, inhibit growth and development, and decrease metabolic activity and survival in the Pacific oyster, *Crassostrea gigas* [14,15].

Additionally, since 1960, global tidal dynamics have intensified, contributing to a bimodal salinity pattern characterized by increasing salinity in already high-salinity areas and decreasing salinity in low-salinity areas [16]. Intense rainfall has also altered estuarine and coastal salinity regimes, causing significant economic losses to *R. philippinarum* aquaculture [6,17], as it cannot maintain normal metabolic functions at salinities below 15 ppt or salinities below 5 ppt [7]. In contrast, long-term exposure to hyposaline conditions appears to have minimal impact on the survival of *M. mercenaria* [18]. Despite numerous studies examining the effects of heat or hyposaline stress on the physiological and immune functions of these two species, none have investigated differences in their responses under the same environmental fluctuations. These differences may determine which species gains a competitive advantage under future climate change scenarios.

The physiological parameters of shellfish, such as filtration, oxygen consumption, and ammonia excretion rates, serve as important indicators of welfare and health status and can be used to evaluate the impact of environmental stressors. Among these, filtration rate is particularly indicative of feeding activity and overall health. Studies on various bivalve species—including the golden mussel *Limnoperna fortunei* [19], the wrinkled rock-borer *Hiatella arctica* (L.) [20], and the blood clam *Scapharca broughtonii* [21]—have shown that filtration rate increases as temperature rises to an optimal, after which it declines. Similarly, oxygen consumption rates increase significantly as temperature rises from 15 to 35 °C [14], while ammonia excretion rate is an indicator of protein metabolic activity since ammonia-nitrogen is the primary product of nitrogen metabolism in shellfish [22].

Numerous enzymes and functional proteins play essential roles in shellfish’s response to environmental stress. Fatty acid desaturases (FADs) are key enzymes in the polyunsaturated fatty acid synthesis pathway and are present in most aquatic animals, including bivalves [23]. In bivalves, FADs participate in multiple physiological processes, such as immune responses, thermal regulation, growth and development, reproduction, regulating cellular homeostasis, and facilitating thermal tolerance [24]. Also, heat shock proteins (HSPs), which function as molecular chaperones, are vital for maintaining cellular homeostasis and environmental stress protection in shellfish [25]. They comprise several families, including HSP20, HSP70, and HSP90, each exhibiting distinct expression patterns in response to fluctuations in temperature and salinity. Among these, HSP70 is one of the most conserved and important families, with 88 *HSP70* genes identified within the *C. gigas* genome alone—all crucial for safeguarding cells against environmental stress [26]. Related studies have also been conducted on the Japanese scallop *Mizuhopecten yessoensis* [27]. Notably, exposure to salinity and heat stress also induces the production of reactive oxygen species (ROS) in bivalves, which in turn activate antioxidant enzymes such as superoxide dismutase (SOD) [28], which protect cells and help maintain homeostasis by mitigating oxidative stress. Therefore, SOD activity serves as a critical indicator of the physiological and immune status of shellfish [29]. Finally, Na^+^/K^+^-ATPase (NKA) functions as a transmembrane ion transporter, driving the active transport of ions across cell membranes and playing a crucial role in osmoregulation [30]. Under salinity and heat stress, mollusks must effectively control both osmoregulation and energy metabolism, and NKA is pivotal in maintaining intracellular and extracellular osmotic balance, signal transduction, and substance transport [31].

This study aims to comparatively investigate the physiological, biochemical, and gene expression responses of *R. philippinarum* and *M. mercenaria* under heat and hyposaline stress. Specifically, it seeks to examine whether the two species differ in their tolerance and response patterns to these stressors, and to identify which species exhibits greater physiological plasticity. The findings will provide a mechanistic basis for evidence-based selection and aquaculture management of economically important marine bivalves.

## 2. Materials and Methods

### 2.1. Collection and Acclimation of Experimental Clams

**Figure 1:**
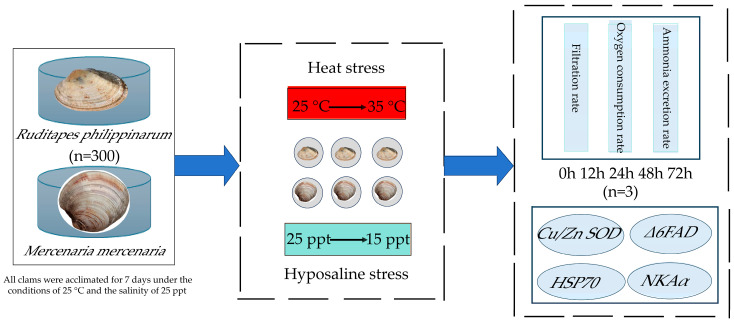
Experimental design. *Cu*/*Zn* SOD: copper-zinc superoxide dismutase; Δ6FAD: Delta-6 fatty acid desaturase; HSP70: heat shock protein 70; NKAα: Na^+^/K^+^-ATPase α-subunit isoform.

More than 600 adult *R. philippinarum* and *M. mercenaria* were collected from the clam-farming ponds in Wenzhou, Zhejiang Province, in August 2023. The Manila clam is generally oval-shaped, with fine radial ribs, and colors and patterns on the shell surface (Figure 1). The shell of the hard clam is grayish-white or purplish-brown, triangular-ovoid with a prominent apex and fine growth rings. The clams were transported in an insulated container to the Ninghai Research Center of East China Sea Fisheries Research Institute, where they were temporarily acclimated in a concrete aquaculture tank (4 m × 8 m × 1.8 m). The average shell length and wet weight of *R. philippinarum* were 36.65 ± 3.22 mm and 10.28 ± 1.96 g, respectively, while those of *M. mercenaria* were 41.23 ± 3.06 mm and 20.63 ± 2.78 g, respectively. The clams were acclimated in natural seawater at a salinity of 25 ppt, and a temperature of 25 ± 0.5 °C, and half of the seawater was replaced every three days. The clams were fed in the morning and evening with a 1:1 mixture of marine algae (*Platymonas helgolandica* var. *tsingtaoensis* and *Chaetoceros muelleri*), at a rate of approximately 300 L per feeding and concentrations of approximately 1 × 10^7^ and 3 × 10^7^ cells/mL. The microalgae used in this experiment were cultured at 22–26 °C in sterilized seawater using a scale-up from 40 L to 300 L plastic drums.

### 2.2. Experimental Design

After one week of acclimation, 300 individuals of *R. philippinarum* and 300 of *M. mercenaria* were divided equally into three 500 L tanks, with 100 individuals per tank. All tanks were subjected to the same experimental conditions. Assuming that temperatures of 35 °C pose a survival risk for Manila clams (unpublished data), the water temperature was gradually increased from 25 °C to 35 °C over 6 h, and subsequently maintained at 35 ± 0.5 °C with a DL336162 thermostat (Deli, Ningbo, China) and heating rods (Sunsun, Zhoushan, China). The experiment commenced once the target temperature of 35 °C was reached. To investigate the effects of hyposaline stress, each species was also placed in three separate 500 L tanks (100 individuals per tank), with an initial salinity of 25 ppt. Freshwater was gradually added over 6 h to reduce salinity to 15 ppt, and the experiment began once the salinity stabilized at this level.

During these experiments, half of the seawater in each tank was replaced daily with seawater maintained at 35 °C or at a salinity of 15 ppt, depending on the treatment. Filtration rate (*F*), oxygen consumption rate (*R*), and ammonia excretion rate (*E*) were measured at 0, 12, 24, 48, and 72 h on each clam. Following the physiological measurements, clams were dissected, and the soft tissues were dried at 65 °C for 24 h before determining the dry mass. At each time point, three clams were randomly selected, and the hepatopancreas was excised and immediately placed in cryogenic vials containing 2 mL of RNA preservation solution. Samples were subsequently stored at −80 °C for gene expression analysis. Clam mortality was assessed in a separate trial with an additional 300 clams maintained in three independent tanks (set up as before).

### 2.3. Physiological Indicator Measurement

Filtration rate was defined as the volume of water cleared of *C. muelleri,* which is retained with 100% efficiency by the gills, per unit time [32]. Each clam was individually placed in a 4 L experimental aquarium containing well-mixed seawater supplemented with *C. muelleri* at an initial concentration of approximately 10,000 cells/mL. During the experiment, water temperature was maintained at 35 °C or a salinity of 15 ppt. Each measurement lasted 30 min and was conducted in triplicate, with empty tanks containing seawater serving as controls. Upon completion, a 1 L water sample was collected from each tank to determine the chlorophyll a (*Chl a*) concentration in both the control and experimental tanks, following the method described by Porra [33]. Briefly, water samples were filtered through 0.45 μm cellulose ester (aqueous) filter membranes (Shanghai Xingya Purification Material Factory, Shanghai, China). The membranes were frozen at −20 °C for 1 h and then thawed at room temperature for 20 min; this freeze–thaw cycle was repeated three times under light-protected conditions to ensure complete cell lysis. Each filter membrane was then placed in a centrifuge tube containing 10 mL of methanol and subjected to dark extraction at 4 °C for 24 h. Next, samples were vortexed and centrifuged at 3500 rpm for 10 min to facilitate *Chl a* extraction. The supernatant was transferred to a quartz cuvette with a 1 cm pathlength for spectrophotometric analysis, and absorbance (*E*) was measured sequentially at 750 nm, 665 nm, and 652 nm. The *Chl a* concentration was calculated as follows:

Concentration of *Chl a* = [16.29 × (*E*_665_ − *E*_750_) − 1.58 × (E_652_ − *E*_750_)] × *V_methanol_*/(*V_sample_* × δ)

where *E*_750_, *E*_665_, and *E*_652_ represent the absorbance of the extract solution at 750 nm, 665 nm, and 652 nm, respectively; *V_methanol_* is the volume (mL) of the methanol extract; *V_sample_* is the volume (mL) of the filtered water sample; and δ is the optical path length (cm) of the cuvette.

Filtration rate was calculated using Coughlan’s formula [32]:

*F* = *V* × ln(*C*_0_/*C_t_*)/(*m* × *t*)

where *V* is the seawater volume in the tank at the time of sampling; *C*_0_ and *C_t_* are the *Chl a* concentrations (mg/L) in the control and experimental tanks at time *t*, respectively; *m* is the dry weight of the soft tissue of the clams (g); and *t* indicates the duration of the experiment (h).

Respiration rate was measured in a 0.56 L respiration chamber, in which each clam was placed inside a mesh bag and suspended to allow free movement without interference. The chambers were sealed with rubber stoppers, and a magnetic stirrer was used to ensure thorough mixing of the seawater. Dissolved oxygen concentration in the chamber was continuously monitored using a Leici JPBJ-609L oxygen meter equipped with a DO-968-HC oxygen electrode (Shanghai INESA Scientific Instrument Co., Ltd., Shanghai, China). The electrode was calibrated using a two-point method, in a zero-oxygen environment (5% anhydrous sodium sulfite solution) and in air-saturated seawater. Calibration was performed once before the start of the experiment, and the water temperature was maintained at 35 ± 0.5 °C. Measurements commenced once the clams opened their shells and continued for 1 h, during which the dissolved oxygen concentration in the respiration chambers decreased by about 2 mg O_2_/L. Oxygen readings were collected at 5 min intervals, yielding 12 measurements per session. Each measurement was conducted in triplicate, with an additional empty chamber containing seawater serving as the control. At the end of the experiment, water samples were collected from each respiration chamber to determine ammonia concentration using the phenol-hypochlorite method [34]. In addition, the wet mass of each clam in the respiration chamber was recorded.

Oxygen concentration was plotted as a function of time and represented by a linear regression line. *R* (mg O_2_/g/h) was calculated using the following formula:

*R* = *b* × *V*/*W*

where *b* is the slope of the regression line, *V* is the water volume in the respiration chamber (L), and *W* is the wet mass of the clams in the respiration chamber (g).

Excretion rate (mg/g/h) was calculated using the following equation:

*E* = (*N*_t_ − *N*_0_) × *V* ÷ (*m* × *t*)

where *N*_t_ and *N*_0_ are the NH_4_^+^-N concentrations (μg/L) in the experimental and control chambers at the end of the experiment, respectively; *V* is the water volume in the respiration chamber (L); *W* is the wet mass of the clams (g); and *t* is the duration of the experiment (h).

### 2.4. RNA Extraction and cDNA Synthesis

Total RNA was extracted from clam tissue samples using a total RNA extraction kit (Biospin, Hangzhou, China) following the manufacturer’s protocol. Briefly, after thawing, 50 mg of each hepatopancreas was placed in a homogenization tube containing 1 mL of Trizol reagent. The tissue was homogenized for 30 s, mixed thoroughly, and allowed to stand at room temperature for 10 min. Subsequently, 200 μL of RNA separation solution was added to each centrifuge tube and the mixture shaken for 15 s, followed by centrifugation at 12,000× *g* for 10–15 min. The upper aqueous phase, containing the RNA, was carefully collected (~500 μL) and mixed with an equal volume of RNA precipitation solution. The mixture was centrifuged at 4 °C at 12,000× *g* for 10 min, and the resulting pellet was washed with 1 mL RNA washing solution at 4 °C, and centrifuged at 7500× *g* for 5 min. The RNA purity and concentration were assessed using a NanoDrop ND-2000C spectrophotometer (Thermo, Waltham, MA, USA). Samples with an OD 260/280 value between 1.8 and 2.1 were selected for downstream experiments, while the remaining samples were stored at −80 °C. Reverse transcription to cDNA was conducted using the Prime Script™ RT Master Mix kit (TaKaRa, Dalian, China).

### 2.5. Gene Transcription Analysis

Relative gene expression levels were quantified using a quantitative PCR kit (Foregene Easy™ Mix-SYBR Green I, Chengdu, China) on a QuantStudio Real-time PCR system (Thermo, MA, USA). The 20 µL reaction mixture contained 2 μL cDNA (10 ng), 0.4 μL forward primer (200 nM), 0.4 μL reverse primer (200 nM), 10 μL TBGreen, and 6 μL ddH_2_O. The thermal cycling program followed an initial denaturation at 95 °C for 5 min, followed by 35 cycles of denaturation at 95 °C for 30 s, annealing at 55 °C for 30 s, and extension at 72 °C for 1 min. Primers were designed using Primer Premier 5 software based on gene sequences obtained from the NCBI database for *R. philippinarum* and *M. mercenaria* (*Cu*/*Zn SOD*, *HSP70*, Δ*6FAD*, and *NKA α-subunit*), with *EF-1α* serving as the internal reference gene [35,36] (see Table 1 for primer sequences and gene sequence IDs). Since *Cu*/*Zn SOD* primarily scavenges superoxide anions generated by the mitochondrial respiratory chain, and *Cu*/*Zn SOD* removes O_2_^−^ from the cytoplasm and extracellular environment, *Cu*/*Zn SOD* was selected as the target gene in the present study (it has been reported that the expression of *Cu*/*Zn SOD* is mainly distributed in the hepatopancreas of *Meretrix meretrix* [37]). All primers were synthesized by Sangon Biotech (Shanghai) Co., Ltd. (Shanghai, China). Relative gene expression was calculated using the 2^−∆∆CT^ method [38].

### 2.6. Statistical Analysis

Data analysis was performed in IBM SPSS Statistics (25.0), and all results are expressed as mean ± SD. Before performing parametric tests, the assumptions of normality were tested using the Shapiro–Wilk test and homogeneity of variances were evaluated using Levene’s test (median-based), and normality was checked. As the data met these assumptions, differences among multiple time points were evaluated using one-way analysis of variance (ANOVA). When significant overall effects were observed, pairwise comparisons were performed with *p*-values adjusted by the Bonferroni post hoc test (*p* < 0.005). For comparisons involving only two groups, an independent samples *t*-test was applied. The two-way analysis of variance was performed to evaluate the effects of time, species, and interaction on physiological parameters and gene expression. Statistical significance was defined as *p* < 0.05. Graphical representations were generated in GraphPad Prism 10.1.0.316.

## 3. Results

### 3.1. Physiological and Gene Expression Responses to Heat Stress

**Figure 2:**
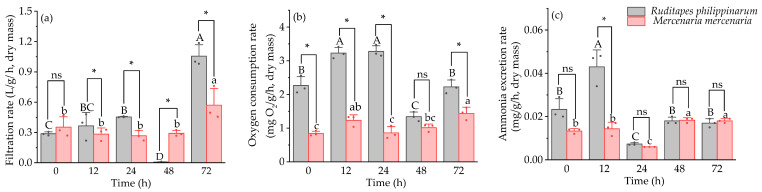
Filtration rate (**a**), oxygen consumption rate (**b**), and ammonia excretion rate (**c**) of *R. philippinarum* and *M. mercenaria* under heat stress. Different letters indicate significant differences among different time points for *R. philippinarum* (uppercase letters)/*M. mercenaria* (lowercase letters) (*p* < 0.05). Asterisk (*) represents a significant difference (*p* < 0.05) between *R. philippinarum* and *M. mercenaria* at each sampling time. ns means non-significant (*n* = 3).

Under heat stress, the cumulative mortality of *R. philippinarum* was significantly higher than that of *M. mercenaria* at 12, 24, 48, and 72 h (*p* < 0.05) (Table 2). Filtration rate (F) was also significantly higher in *R. philippinarum* compared to *M. mercenaria* at 12, 24, and 72 h (*p* < 0.05), while *M. mercenaria* exhibited higher filtration and oxygen consumption rates at 48 h (Figure 2a,b). Ammonia excretion rate was significantly greater in *R. philippinarum* compared to *M. mercenaria* at 12 h (*p* < 0.05), with no significant differences observed at the other time points (Figure 2c). Statistical analysis indicated significant interactive effects of time and species on filtration rate, oxygen consumption rate, and ammonia excretion rate (*p* < 0.05).

**Figure 3:**
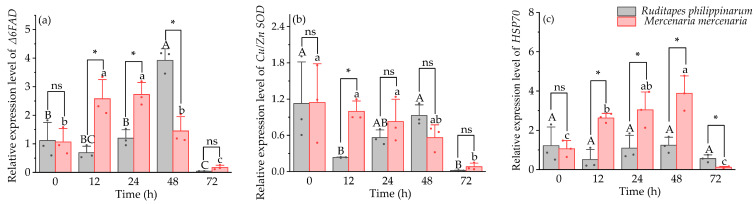
Expression level of Δ*6FAD* (**a**), *Cu*/*Zn SOD* (**b**), and *Hsp70* (**c**) in *R. philippinarum* and *M. mercenaria* under heat stress. Different letters indicate significant differences among different time points for *R. philippinarum* (uppercase letters)/*M. mercenaria* (lowercase letters) (*p* < 0.05). Asterisk (*) represents a significant difference (*p* < 0.05) between *R. philippinarum* and *M. mercenaria* at each sampling time. ns means non-significant (*n* = 3).

Under heat stress, Δ*6FAD* gene expression in *M. mercenaria* was significantly higher than in *R. philippinarum* (*p* < 0.05) at 12 h and 24 h, whereas this trend was reversed at 48 h (*p* < 0.05) (Figure 3a). At 12 h, *Cu*/*Zn SOD* expression in *M. mercenaria* was significantly higher compared to *R. philippinarum* (*p* < 0.05), with no significant differences observed at the other time points (Figure 3b). Expression of *HSP70* was significantly elevated in *M. mercenaria* relative to *R. philippinarum* at 12 h, 24 h, and 48 h (*p* < 0.05) (Figure 3c). Significant interactive effects of time and species were observed for Δ*6FAD* and *HSP70* expression (*p* < 0.05), whereas no significant effect was detected for *Cu*/*Zn SOD* expression (*p* > 0.05).

### 3.2. Physiological and Gene Expression Responses to Hyposaline Stress

**Figure 4:**
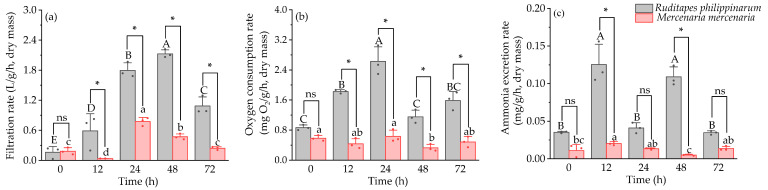
Filtration rate (**a**), oxygen consumption rate (**b**), and ammonia excretion rate (**c**) of *R. philippinarum* and *M. mercenaria* under hyposaline stress. Different letters indicate significant differences among different time points for *R. philippinarum* (uppercase letters)/*M. mercenaria* (lowercase letters) (*p* < 0.05). Asterisk (*) represents a significant difference (*p* < 0.05) between *R. philippinarum* and *M. mercenaria* at each sampling time. ns means non-significant (*n* = 3).

Under hyposaline stress, there was no significant difference in mortality rates between the two species, although the filtration rate and oxygen consumption rate of *R. philippinarum* were significantly higher compared to *M. mercenaria* at 12, 24, 48, and 72 h (*p* < 0.05) (Figure 4a,b). Ammonia excretion rate was significantly greater in *R. philippinarum* compared to *M. mercenaria* at 0, 12, and 48 h (*p* < 0.05) (Figure 4c). Statistical analysis indicated significant interactive effects of time and species on filtration rate, oxygen consumption rate, and ammonia excretion rate (*p* < 0.05).

**Figure 5:**
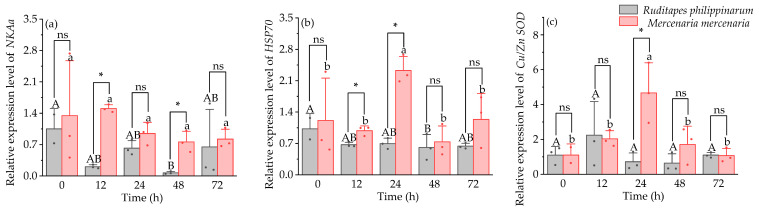
Expression level of *NKAa* (**a**), *HSP70* (**b**), and *Cu*/*Zn SOD* (**c**) genes in *R. philippinarum* and *M. mercenaria* under hyposaline stress. Different letters indicate significant differences among different time points for *R. philippinarum* (uppercase letters)/*M. mercenaria* (lowercase letters) (*p* < 0.05). Asterisk (*) represents a significant difference (*p* < 0.05) between *R. philippinarum* and *M. mercenaria* at each sampling time. ns means non-significant (*n* = 3).

Under hyposaline stress, *NKA* expression was significantly higher in *M. mercenaria* than in *R. philippinarum* at 12 h and 48 h (*p* < 0.05) (Figure 5a). Expression of *HSP70* was significantly higher in *M. mercenaria* than in *R. philippinarum* at 12 and 24 h (*p* < 0.05) (Figure 5b). At 24 h, *Cu*/*Zn SOD* expression was significantly greater in *R. philippinarum* compared to *M. mercenaria* (*p* < 0.05) (Figure 5c). Significant interactive effects of time and species were observed for Δ*6FAD* and *Cu*/*Zn SOD* expression (*p* < 0.05), whereas no significant effect was detected for *NKAa* expression (*p* > 0.05).

## 4. Discussion

This study focused on the temporal effect of heat and hyposaline stresses on two bivalves to reveal and compare trends in the stress parameters. Similar designs have also been adopted and recognized in previous studies on heat and saline stress and the physiological responses of bivalves [39,40].

### 4.1. Differences in Responses to Heat Stress

Temperature, as a prominent environmental stressor, exerts a significant influence on the physiology of filter-feeding bivalves. Shellfish modulate filtration rate by regulating the rhythmic movement of the gill filament cilia and can increase ciliary beating frequency in response to elevated temperatures [41]. This may account for the marked increase in filtration rates in both species under elevated temperatures. Furthermore, the reduced viscosity of seawater at higher temperatures may support these enhanced filtration rates [42]. This increase will subsequently decline beyond optimal temperature conditions [19,21]. The filtration rate of *R. philippinarum* decreased significantly at 48 h, suggesting that 35 °C may exceed the maximum tolerable temperature. Similarly, *R. philippinarum* suffers mortality when water temperatures exceed 34.5 °C (unpublished data). In contrast, *M. mercenaria* exhibited an increased filtration rate at 48 h, indicating that its upper thermal threshold may be higher than 35 °C.

Elevated temperatures enhance the functional activity of tissues and organs by accelerating biochemical reactions. Consequently, in poikilothermic species, respiration and metabolic rates increase, coupled with elevated oxygen consumption and ammonia-nitrogen excretion [43]. Bivalves require greater energy expenditure to sustain normal physiological functions under heat stress, as was seen by enhanced metabolic levels in two bivalve species (*Mulinia edulis* and *M. chilensis*) [44]. The significant increase in the metabolic rate of *R. philippinarum* here aligns with these findings [14]. However, prolonged exposure to heat stress may impair the physiological performance of clams, explaining the ultimate decrease in metabolic rate. Similar observations have been reported for the Japanese scallop, in which both oxygen consumption and ammonia-nitrogen excretion rates increased significantly compared to the control group [45]. Current results also suggest that *M. mercenaria* exhibits greater thermal tolerance and regulatory capacity under heat stress, enabling the maintenance of cellular metabolic homeostasis. In contrast, *R. philippinarum* exhibited a greater magnitude of metabolic changes and higher mortality rates under prolonged heat stress.

Fatty acid desaturase is a rate-limiting enzyme in the biosynthetic pathway of highly unsaturated fatty acids (HUFAs) [46]. The expression levels of Δ*6FAD* in *R. philippinarum* and *M. mercenaria* were similar, but more pronounced in *R. philippinarum*. This suggests that *R. philippinarum* is more sensitive to temperature fluctuations. Under heat stress, *M. edulis* and *C. virginica* remodel their membrane phospholipids to increase the proportion of unsaturated fatty acid 20:4n-6 in the gills and digestive glands [46]. This response requires a significant upregulation of Δ*6FAD*, also observed in the current study. However, the proportion of 22:6n-3 and 20:5n-3 decreased in the tissues of *M. edulis* and *C. virginica* [47]. Although gene expression is often correlated with phenotype, changes in gene expression do not necessarily translate directly into functional changes. Post-translational modifications of proteins, such as methylation, phosphorylation, ubiquitination, and acetylation, also play a significant role in phenotype [48]. Therefore, the mechanisms by which heat stress influences fatty acid metabolism in shellfish need further investigation.

Abrupt temperature fluctuations can trigger excessive production of ROS and oxidative damage [49] and, under severe conditions, physiological dysfunction or mortality. Shellfish typically exhibit increased SOD enzyme activity and gene expression levels in response to thermal stress. Banni et al. [50] observed that *SOD* gene expression was initially significantly upregulated in the Mediterranean mussel *Mytilus galloprovincialis* during heat stress at 26 °C, followed by a notable downregulation after 72 h of exposure. In this study, the downregulation of *Cu*/*Zn SOD* gene expression suggests a diminished capacity to scavenge mitochondrial ROS under heat stress, reflecting heat stress that exceeded the tolerance limits of both bivalve species, as indicated by the high mortality rates. Similarly, Liu et al. [51] reported significantly reduced mRNA levels and enzymatic activity of superoxide dismutase in the gills of *Cyclina sinensis* following short- and long-term heat exposure.

Heat stress also induces protein denaturation and aggregation, disrupting organelle integrity and inhibiting essential biological processes [52]. Here, accumulation of HSPs is crucial in protecting cells against protein denaturation [53]. In this study, *R. philippinarum* exhibited a decrease in *HSP70* gene expression, suggesting reduced heat tolerance. Nie et al. [54] also reported decreased *HSP70* expression in *R. philippinarum* following a direct transfer from 22 °C to 30 °C seawater. Likewise, under acute heat stress, *HSP70* expression increased significantly in *M. yessoensis* at 12 h before returning to baseline levels [27].

Notably, *M. mercenaria* exhibited significantly upregulated expression of Δ*6FAD*, *SOD*, and *HSP70* at most time points compared to *R. philippinarum*, suggesting a higher sensitivity to thermal challenge. However, this expression pattern is not directly correlated with physiological sensitivity, as reflected by the oxygen consumption and ammonia excretion rates addressed above. The ability of the hard clam to regulate its metabolism under heat stress enables energy availability for growth. Thus, enhanced SOD and HSPs capacities could improve cellular protection and metabolic homeostasis, contributing to greater physiological stability and thermal tolerance [55,56]. Therefore, *HSP70* expression could serve as a useful molecular indicator for thermal stress in *M. mercenaria*, whereas oxygen consumption and ammonia-nitrogen excretion rates serve as physiological indicators in *R. philippinarum*.

### 4.2. Differences in Response to Hyposaline Stress

Salinity is a critical environmental factor influencing the survival, growth, and physiological metabolism of shellfish. Mortality data from the present study indicate that both species exhibit considerable tolerance to hyposaline stress. Under extremely low salinity, bivalves respond to external osmotic pressure changes by partially or completely closing their shells, thereby decreasing filter-feeding [57]. Studies on the Asian green mussel *Perna viridis* and *C. virginica* have demonstrated that decreasing salinity from 30 to 25, 15, and 10 ppt significantly reduces the filtration rates and increases shell-closing frequency [58]. Thus, the initial decline in filtration rates seen in both species suggests a behavioral-physiological response, where *M. mercenaria* exhibits greater low-salinity tolerance as evidenced by a more stable filtration rate.

Comparatively, *R. philippinarum* showed a higher oxygen consumption rate, signifying greater energetic costs for osmoregulation under salinity stress. Similarly, Maboloc and Villanueva [59] reported a significant increase in oxygen consumption in juvenile *Tridacna gigas* 96 h after acute salinity reductions from 35 to 25 ppt and 18 ppt. This suggests a heightened metabolic demand during osmotic adjustment. In contrast, the relative stability of oxygen consumption in *M. mercenaria* across salinity treatments reflects its superior osmoregulatory capacity. In support of this, Ivanina et al. [60] demonstrated that mitochondrial respiration in isolated *M. mercenaria* gill and mantle cells remained unaffected after 14-day exposures to salinities of 30, 18, and 10 ppt. This points to a robust cellular metabolic resilience to osmotic stress.

Observed significant fluctuations in ammonia excretion rates observed in *R. philippinarum* further underscore the substantial impact of hyposaline stress on shellfish metabolism. Under hypoosmotic conditions, shellfish decrease feeding but increase energy expenditure [61]. Protein catabolism is upregulated to sustain metabolic function, ultimately resulting in elevated amino acid concentrations in the hemolymph and increased ammonia excretion rates [62]. In contrast, the physiological stability of *M. mercenaria* over 72 h highlights its superior homeostatic control under salinity stress.

Most marine mollusks are osmoconformers, with the osmolarity of their extra- and intracellular fluids varying according to environmental salinity [63]. This is energetically costly but necessary to prevent extreme changes in cellular volume [64]. In bivalve mollusks, inorganic ions such as Na^+^, K^+^, and Cl^−^ serve as buffers to mitigate the effects of drastic fluctuations in salinity [65]. It has been reported that the concentrations of Na^+^, K^+^, and Cl^−^ decreased significantly in the hemolymph of *R. philippinarum* under low-salt stress [66], indicating that this species responds to salinity fluctuations by adjusting ion concentrations. Unlike the oxygen consumption rate, NKAα gene expression in *R. philippinarum* decreased significantly at 48 h, possibly reflecting a response to decreased intracellular ion concentrations to lower the cation transport capacity of the cell membrane. Other ion transport channels may also contribute to maintaining ionic balance. Consistently, Liu et al. [39] reported that NKA activity in *R. philippinarum* decreased significantly when salinity was reduced from 30 to 10 ppt. Similarly, in *Haliotis discus hannai*, hyposaline stress significantly suppressed both NKA activity and gene expression. Western blot analysis confirmed a marked reduction in NKAα protein levels, and transcriptomic profiling revealed significant changes in genes associated with ion transport [66]. Again, the stable NKA expression in *M. mercenaria* indicates a superior intrinsic capacity for osmotic homeostasis under hyposaline stress. Notably, the NKA enzyme activity of *M. mercenaria* was unaffected when salinity decreased from 30 to 18 ppt but declined markedly at 10 ppt [60]. Isoform switching of NKA has been shown to play a critical role in salinity adaptation in euryhaline fish [67], but research on Na^+^/K^+^-ATPase in mollusks remains limited. Future studies should investigate the functional role of NKA in molluscan salinity adaptation through integrated approaches involving enzyme activity assays and immunohistochemical localization.

Hyposaline stress can induce osmotic imbalance, increasing ROS production [28]. Nie et al. [68] reported that *SOD* expression in wild *R. philippinarum* increased significantly when salinity was reduced to 15 ppt. However, prolonged exposure to severe stress can disrupt intracellular homeostasis and suppress *SOD* activity [69]. The stable *Cu*/*Zn SOD* expression observed in both species implies a shared baseline transcriptional resilience to salinity fluctuations, with *M. mercenaria* exhibiting a temporally constrained antioxidant response. This pattern may reflect post-transcriptional regulatory mechanisms, including isoform switching and post-translational modifications (e.g., phosphorylation or ubiquitination), to modulate SOD activity independently of mRNA abundance.

Unlike under heat stress, *HSP70* transcription in *R. philippinarum* remained relatively stable under hyposaline conditions. In contrast, Nie et al. [54] observed significant fluctuations in *HSP70* gene expression in *R. philippinarum* when salinity was abruptly reduced from 32 to 15 ppt, suggesting that the method and rate of exposure can influence gene expression patterns. The progressively increased *HSP70* gene expression in *M. mercenaria* highlights that under hyposaline stress, *M. mercenaria* can protect cells from salinity-induced damage by correcting misfolded proteins and eliminating abnormal proteins through elevated *HSP70* gene expression [70,71]. This is consistent with findings in *P. martensii* [72].

### 4.3. Limitations of the Study

Animal metabolic scaling typically follows an allometric power law, expressed as *R* = *aW^b^*, where *R* represents the metabolic rate, *W* is body mass, *a* is the scaling coefficient, and *b* is the scaling exponent. Since most reported exponents (*b*) are significantly less than 1, mass-specific metabolic rate decreases with increasing body size for a given species [73]. Thus, body size is a key factor determining physiological capabilities across species, strongly influencing energy distribution strategies and life-history trade-offs [74]. Typically, adult Manila clams have a shell length of 3–4 cm, whereas adult hard clams reach 5–6 cm. These differences in body size are inherently part of the comparison, although all physiological parameters in this study were standardized and expressed per unit dry tissue mass. This normalization eliminates the confounding effects of interspecific differences in body size. Thus, the observed differences in stress tolerance can be considered ecologically and physiologically relevant.

Finally, in this study, control groups at constant salinity and temperature were not established for the two species for several reasons. However, previous studies have been conducted on each species separately, with control groups indicating no significant changes. For example, Kim et al. [7] found through 271.5 h of continuous measurements that, under conditions of ‘constant’ salinity (31.2 to 31.5 ppt), a temperature of 20.5 ± 0.1 °C, and constant darkness, the oxygen consumption rate of *R. philippinarum* exhibited a circatidal rhythm at 12.6 h intervals. Liu et al. [75] reported that the oxygen consumption rate and ammonia excretion rate of Manila clams remained stable for seven days under stable conditions (24 °C, 30 ppt, and 6 mg/L dissolved oxygen). Also, no significant variations were observed in the oxygen consumption rate of *M. mercenaria* after exposure to 22 °C (control) for 15 weeks [76]. Therefore, the changes in parameters observed in the two bivalves in this study are safely attributable to changes in temperature and salinity.

## 5. Conclusions

This study showed that *R. philippinarum* exhibited significant metabolic fluctuations during the initial phase of heat stress, characterized by increased filtration, oxygen consumption, and ammonia excretion rates. In contrast, *M. mercenaria* demonstrated smaller metabolic fluctuations and maintained relative physiological stability. Expression levels of Δ*6FAD*, *HSP70*, and *Cu*/*Zn SOD* were generally higher in *M. mercenaria* than in *R. philippinarum*. Under hyposaline stress, the feeding behavior and metabolic activity of *R. philippinarum* varied, whereas *M. mercenaria* maintained relative stability in oxygen consumption and ammonia excretion rates. Notably, *M. mercenaria* exhibited elevated expression of *NKA* and *HSP70* compared to *R. philippinarum*. By maintaining feeding and metabolic stability while rapidly upregulating key stress-response genes, *M. mercenaria* demonstrates enhanced physiological flexibility to environmental changes. These findings provide valuable insights into the mechanisms underlying bivalve responses to environmental stress. Results offer theoretical guidance for aquaculture species selection and management.

## Figures and Tables

**Table 1:** Sequences of primers for qPCR.

Primer Name	Gene Sequence ID Number	Primers Sequence (5′-3′)	Amplicon Sizes	Efficiency (%)
*Cu*/*Zn* *SOD* of *R. philippinarum*	XM_060747330.1	F: AACACTTGTCGTTCACGCTG	82	107
		R: TCCTCCAGCATTTCCAGTCG		
*Cu*/*Zn* *SOD* of *M. mercenaria*	XM_053542271.1	F: CACGACAATACACCGTCCCA	132	103
		R: CAAGGTCGGAAAGAGCCAGA		
*HSP70* of *R. philippinarum*	XM_060747667.1	F: TGAGCGAGCAAAGAGAACACTGTC	137	107
		R: TACCACGGAACAAGTCGCATTAAG		
*HSP70* of *M. mercenaria*	XM_045351428.2	F: GAGCTCCACCAGCTTGATAGAGT	92	109
		R: GGCTGCTAAGGACGAGTATGAAC		
Δ*6FAD* of *R. philippinarum*	XM_060703940.1	F: CGATGTTGTCCTGTGGTGG	91	95
		R: TTCGTGGAGTAGTTTCGTCTTC		
Δ*6FAD* of *M. mercenaria*	XM_045334470.2	F: TCCGGGTTTCAAAGAGGAAGAG	131	98
		R: AGAGCAGTGTTGAGGCACTT		
*NKAα* of *R. philippinarum*	XM_060727913.1	F: TCCAACTCCACCTACAGCCGATC	100	92
		R: CTTCTTCCTGGTCTGCCTTGAACTC		
*NKAα* of *M. mercenaria*	XM_053552503.1	F: TATGCCTGCCTTTCCGGTTT	82	109
		R: GCTTGAGCAATGTATCAGAGCC		
*EF-1α* of *R. philippinarum*	XM_060695428.1	F: GAATGGTTGTTACCTTTGCTCC	214	104
		R: ACGATGACCTGGGCATAGA		
*EF-1α* of *M. mercenaria*	XM_045326554.2	F: AGTCGGTCGAGTTGAAACTGGTGT	114	101
		R: TCAGGAAGAGACTCGTGGTGCATT		

**Table 2:** Mortality (%) of *R. philippinarum* and *M. mercenaria* during heat and hyposaline stress.

Time (h)	Heat Stress		Hyposaline Stress	
	*R. philippinarum*	*M. mercenaria*	*R. philippinarum*	*M. mercenaria*
0	0	0	0	0
12	4.33 ± 2.08 *	0.33 ± 0.58	0	0
24	13.33 ± 5.13 *	2.67 ± 1.15	0.67 ± 0.58	0.33 ± 0.58
48	33.67 ± 4.04 *	10.00 ± 2.00	1.33 ± 0.58	0.67 ± 0.58
72	70.67 ± 4.04 *	22.67 ± 4.93	1.67 ± 0.58	1.00 ± 0.00

Asterisk (*) indicates a significant difference in the mortality rate between *R. philippinarum* and *M. mercenaria* (*n* = 100, mean ± SD).

## Data Availability

The original contributions presented in this study are included in the article. Further inquiries can be directed to the corresponding author.

## Abbreviations

The following abbreviations are used in this manuscript:

*Chl a*	Chlorophyll a
FADs	Fatty acid desaturases
HSPs	Heat shock proteins
SOD	Superoxide dismutase
NKA	Na^+^/K^+^-ATPase
